# Aortic Valve Replacement for Infective Endocarditis After Presternal Esophageal Reconstruction

**DOI:** 10.1016/j.atssr.2023.02.003

**Published:** 2023-02-20

**Authors:** Ryo Taguchi, Ryosuke Kowatari, Hanae Sasaki, Masahito Minakawa

**Affiliations:** 1 Department of Thoracic and Cardiovascular Surgery, Hirosaki University School of Medicine, Hirosaki, Aomori, Japan

## Abstract

A 57-year-old man with dyspnea was admitted to our hospital and diagnosed with infective endocarditis of the aortic valve. He had undergone subtotal esophagectomy with neoesophageal reconstruction from the anterior sternum for esophageal cancer 6 years previously. Progressive heart failure and multiple cerebral infarctions warranted emergency aortic valve replacement by the right thoracotomy approach, which avoided esophageal injury and provided a good surgical view. We suggest that right thoracotomy is a useful option for treating aortic infective endocarditis in patients after esophageal reconstruction with a subcutaneous neoesophagus.

Cardiac surgery after esophageal cancer surgery is challenging because the reconstructed esophagus interferes with the processes performed in the conventional median sternotomy approach. Several studies have introduced an alternative approach for aortic valve replacement (AVR) after esophagectomy in patients with a neoesophagus reconstructed through the retrosternal route.^1^, ^2^, ^3^, ^4^ However, cardiac surgery in cases of a subcutaneous neoesophagus has not been discussed at much length. Herein, we present a case of infective endocarditis (IE) in a patient with a reconstructed neoesophagus through the presternal route that was successfully treated with emergent AVR by a right thoracotomy approach.

A 57-year-old man with dyspnea was transferred to our hospital. He had been diagnosed with antibiotic-resistant pyogenic spondylitis a month earlier. Esophageal cancer had developed at the age of 51 years, and he had undergone subtotal esophagectomy with neoesophagus (pedicled jejunal flap) reconstruction through the anterosternal route (Figure 1A). Transthoracic echocardiography revealed massive vegetation on the aortic valve and severe aortic regurgitation (Figure 2). Enhanced computed tomography revealed a reconstructed pedicled jejunum over the sternum (Figure 1B), and although magnetic resonance imaging did not reveal cerebral hemorrhage, small cerebral infarctions in the occipital lobe were observed. Blood culture revealed the presence of *Enterococcus faecalis*. The patient underwent emergent AVR because of progressive heart failure and multiple cerebral infarctions caused by fragile vegetation.

**Figure 1:**
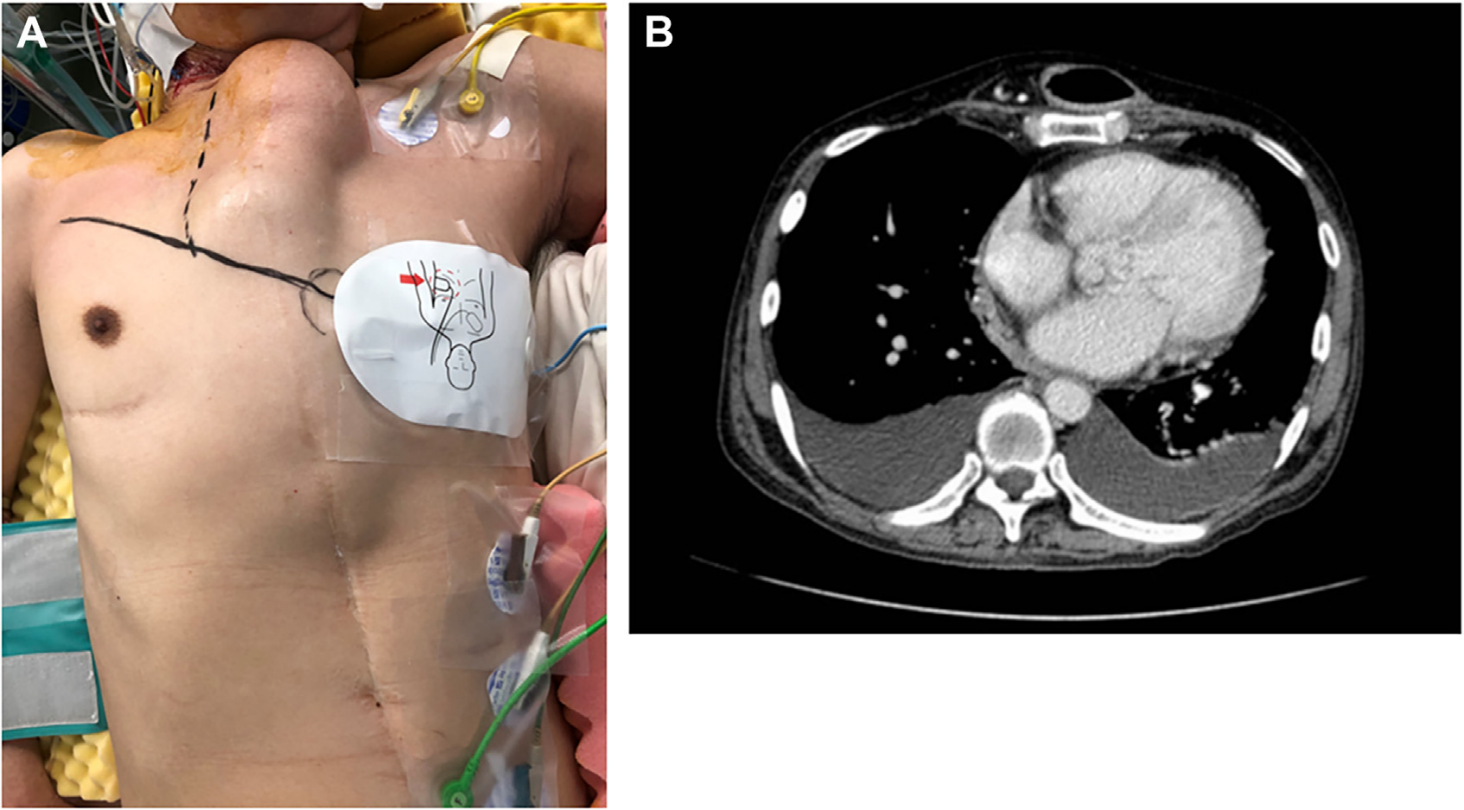
(A) A neoesophagus reconstructed through the anterosternal route is visible in the midline of the trunk. (B) Computed tomography shows that the neoesophagus is located on the sternum.

**Figure 2:**
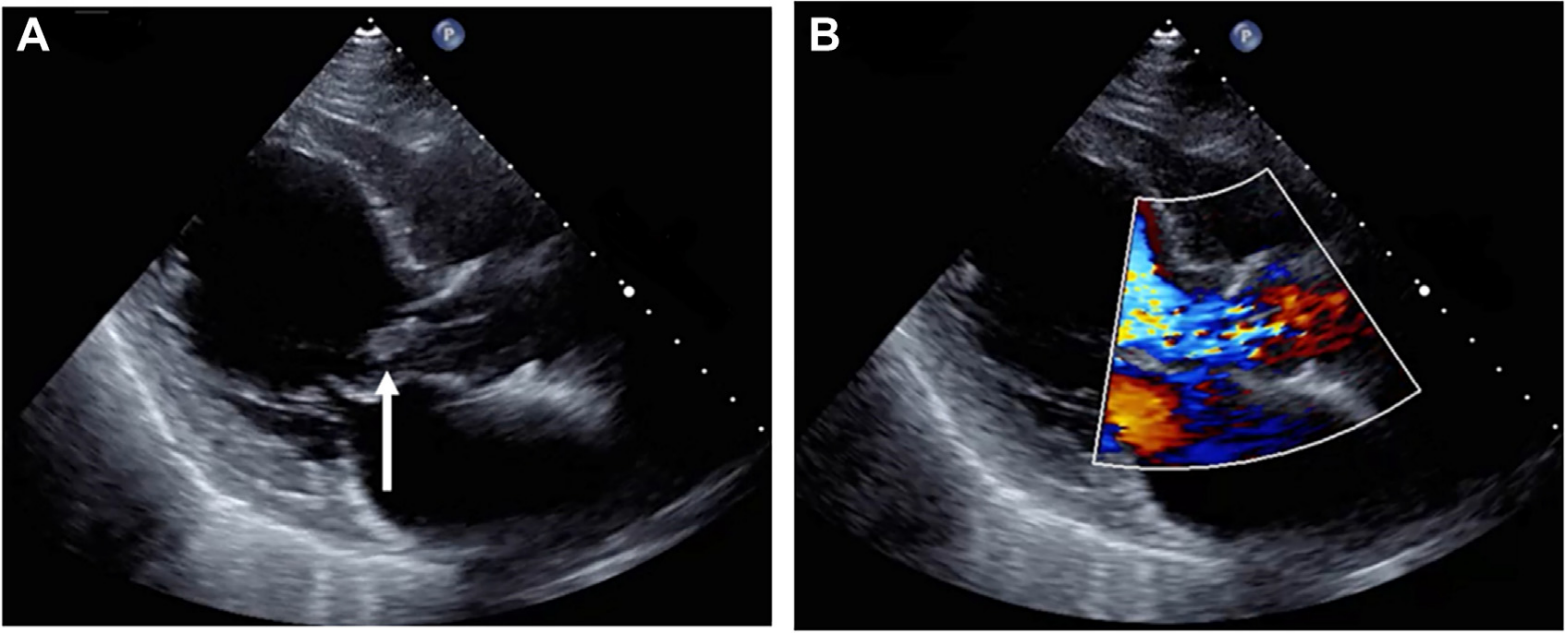
(A) The arrow points to vegetation on the aortic valve. (B) Transthoracic echocardiography demonstrated severe aortic regurgitation.

A summary of the operation is provided in the Video. Under general anesthesia, the patient was positioned on the left side in a 45-degree lateral position following the insertion of a double-lumen endotracheal tube. The approach involved a right anterolateral thoracotomy through the right third intercostal space with a 10-cm skin incision. After splitting of the fourth costal bone and detachment of adhesions in the right thoracic cavity, cardiopulmonary bypass was established with femoral artery and vein cannulation. A venting tube was inserted into the right upper pulmonary vein. The ascending aorta was subsequently cross-clamped and incised, and cardiac arrest was achieved with antegrade selective cardioplegia.

The aortic valve was tricuspid, with vegetation and perforation in each leaflet (Figure 3A). No aortic valvular abscesses were observed. The infected aortic valve was incised, followed by a 21-mm INSPIRIS (Edwards Lifesciences) implantation in the supra-annular position with single interrupted sutures (Figure 3B). Weaning from the cardiopulmonary bypass was uneventful. Cardiopulmonary bypass and cardiac ischemia spanned 176 and 129 minutes, respectively. Tissue culture from the excised cusps revealed *E. faecalis*.

**Figure 3:**
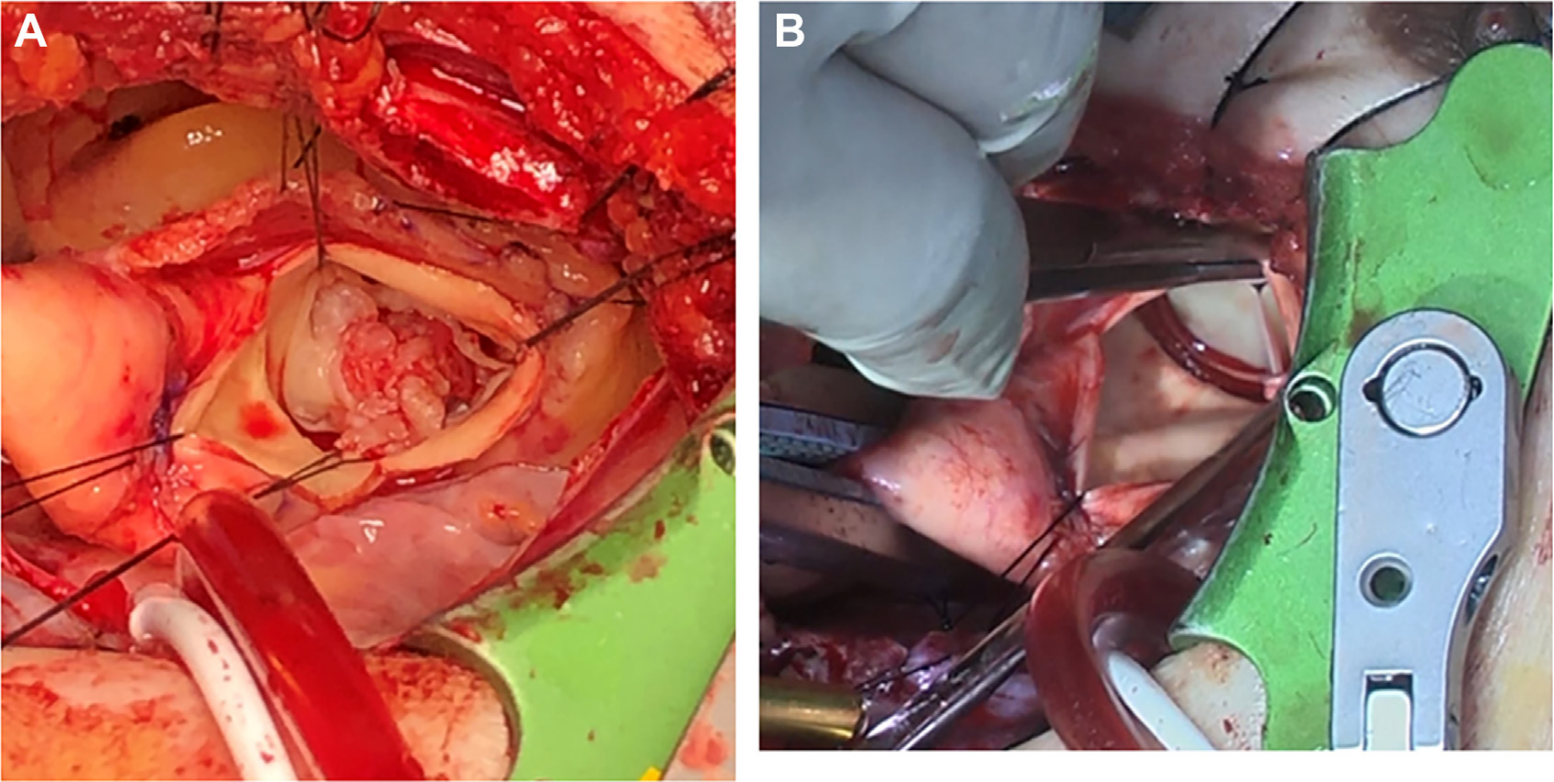
(A) The aortic valve has 3 leaflets, each with vegetation. (B) Aortic valve replacement with a bioprosthetic valve.

Ampicillin (500 mg every 6 hours) and gentamicin (120 mg daily) were administrated for 4 weeks postoperatively. The patient was discharged from our hospital 23 days after surgery without any complications. IE did not recur for 2 years.

## Comment

The right thoracotomy approach to AVR has become more common lately. A retrospective study using propensity score matching reported that the right thoracotomy approach is as safe as median sternotomy and superior in terms of postoperative recovery and respiratory condition.^1^ Fatehi Hassanabad and colleagues^2^ reported the initial 100 cases of right thoracotomy AVR at a single institution and reviewed the benefits, safety, and clinical outcomes of this approach.

Our experience indicates that right thoracotomy is a useful surgical approach in cases requiring aortic valve surgery after esophageal reconstruction with a subcutaneous neoesophagus. Several reports have described AVR performed after esophageal reconstruction.^3^, ^4^, ^5^, ^6^ To avoid injury to the neoesophagus, various approaches, including median sternotomy, right thoracotomy, right parasternal, and left thoracotomy, have been applied to this challenging situation. Unfortunately, there are only a few reports of AVR in patients with a subcutaneous neoesophagus. Inra and coworkers^7^ reported 7 cases of cardiac surgery after extra-anatomic esophageal reconstruction. Even the largest study from Mayo Clinic included only 2 subcutaneous neoesophagus cases: mitral valve replacement through right thoracotomy and an AVR case through median sternotomy. The patient in this case underwent AVR through a right thoracotomy. In the right thoracotomy approach, surgeons can preserve not only the right internal thoracic artery but also the feeding artery of the neoesophagus. We also avoided injury to the esophagus and its surrounding anastomosis site by not choosing a median approach. Fukunaga and coworkers^4^ reported the effectiveness of the right thoracotomy approach in a patient undergoing retrosternal reconstruction. The authors found significant pleural adhesions in the right thorax but noted that dissection of the adhesions was only limited; they were able to reach the pericardial sac and ascending aorta without difficulty. In our case, there were similar severe intrathoracic adhesions; the separation of the fourth rib provided adequate working space and facilitated the dissection of the adhesive part of the right thoracic cavity.

There are also limited reports on right thoracotomy AVR for IE. Guida and colleagues^8^ reported AVR for IE through a small right thoracotomy that allowed good exposure to the aortic valve. This case report is similar to ours in that there was no perivalvular abscess. Right small thoracotomy provides a frontal view of the aortic valve by selecting the appropriate intercostal space so that the viewing angle is parallel to the aortic valve. This approach is considered a reasonable choice because it enables the easy inspection of the aortic valve with a good field of view, enabling débridement and reconstruction in a broader area. Initially, we suspected valvular annular abscess. Temporary cutting of the fourth rib provides a sufficient surgical field, as shown in Figure 3. Perivalvular abscesses were not observed in this case; even if they had been present, this could be handled.

In conclusion, we describe a case of IE in a patient with a reconstructed neoesophagus through the presternal route that was successfully treated with emergent AVR through a right thoracotomy approach. This approach avoided injury to the esophagus and allowed a satisfactory aortic valve view. As shown in this case, a minimally invasive approach makes the operation simpler in some cases; thus, the use of various approaches in individual cases can lead to a successful operation.
